# Self-Aligned Colloidal Lithography for Controllable and Tuneable Plasmonic Nanogaps

**DOI:** 10.1002/smll.201402639

**Published:** 2014-12-15

**Authors:** Tao Ding, Lars O Herrmann, Bart de Nijs, Felix Benz, Jeremy J Baumberg

**Affiliations:** NanoPhotonics Centre, Cavendish LaboratoryJJ Thomson Avenue, Cambridge, CB3 0HE, UK; Department of Materials Science and Metallurgy, 27 Charles Babbage Road, University of CambridgeCB3 0FS, UK

**Keywords:** colloidal lithography, ebeam evaporation, gold films, nanoparticles, SERS

Surface enhanced Raman spectroscopy (SERS) has attracted enormous interest over recent years due to its ability to detect and identify few-to-single molecules that are located in plasmonically enhanced electric fields, so called ‘hot-spots’.[1–3] Upon illumination with a Raman laser, these strongly enhanced electric fields emerge, for example, on roughened gold surfaces or in gold nanoparticle (Au NP) aggregates. However, more uniform nanostructures are required to maintain consistent scattering enhancements, and access only few molecules. A common route towards fabrication of such substrates is the use of rigid molecular linker molecules to form Au NP dimers[4–6] or aggregates[7–9] with controlled interparticle separations. Light-driven mutual electromagnetic coupling between coherent surface charge oscillations in the NPs then creates intense local electric fields in the gaps between the particles.[10] The magnitude of these fields strongly increases with decreasing gap sizes, but the gaps need to remain accessible for analyte molecules.[11] Extremely small reliable gaps on the order of 0.5–3 nm, so called ‘nanogaps’, are therefore ideal for applications of molecular sensing and plasmon-driven chemical reactions.[12]

Several nanofabrication techniques have been developed for the generation of nanogaps, which include e-beam lithography,[13–16] interference lithography,[17] shadow evaporation,[18–24] mechanical break junctions,[25] physical[26–30] and chemical[31,32] templating and others.[33] However, these methods are either complicated and expensive or time-consuming, or lack tuneability of the gap size at small scales. For example most of the top-down approaches such as e-beam lithography and break junctions are only applicable for small scale production with very low efficiency. Shadow evaporation requires masks with nanoholes, which are difficult to make especially over larger areas.[21–24] Physical templates including gratings and nanodome arrays can provide nanogaps between the mesa-like features to generate sub-nanogaps via controlled metal vapor deposition, but fabrication of these templates still requires sophisticated nanofabrication techniques such as extreme ultraviolet interference lithography,[26–28] nanoimprint lithography and reactive ion etching for the molds.[29,30] Although chemical templating approaches are scalable for the production of nanogaps, the ligands in the reaction system always remain in the gap and prevent detection of many types of molecules.[32]

In contrast to physically patterned masks, mask fabrication through bottom-up approaches are more attractive due to their low costs and scalability. In particular, lithography based on colloidal masks (colloidal lithography) combines top-down etching or deposition techniques with bottom-up self-assembly of colloidal particles, thereby providing a cost-effective route towards large scale patterned plasmonic structures.[34] However, most of the common colloidal particles have >50 nm features are either made of polystyrene (PS) or SiO_2_, which always results in gaps with larger dimensions than desired.[35–38] Although there are some clever techniques to generate nanogaps via colloidal lithography, the procedures are often complicated with multistep fabrication procedures.[39–41] Here, we use gold nanoparticles (Au NPs) directly as a colloidal mask for lithography, making it much easier to generate considerably smaller plasmonic gaps. The rationale here is to develop a facile, cost-effective and scalable plasmonic substrate via colloidal lithography that is based on Au NPs, and surprisingly the straightforward approach here has not been reported.

Our method starts by placing Au NPs on the surface of a silicon wafer, followed by e-beam evaporation of an Au film (**Figure** **1**a). The Au NPs here act as a colloidal mask to shadow a ring-shaped region underneath. Therefore nanogaps are generated between the Au NPs and the surrounding Au film, and the size of these gaps can be tuned by adjusting the thickness of the deposited Au film. To test this, 80 nm Au NPs were deposited on a Si wafer via drop casting. The dark field scattering image in Figure 1b shows a green color for the Au NPs on the Si wafer, and the SEM image (Figure 1d) clearly shows the Au NPs on the flat Si wafer with a shadow region below. After evaporating a layer of 30 nm Au (voltage: 10 kV, rate: 1 Å/s, Lesker e-beam evaporator), both the top of the Au NPs and the surface of the Si wafer were covered with Au except the shadowed regions underneath the Au NPs. Hence the Au NPs remain isolated from the Au film and produce nanogaps in-between. The surface roughness of Au films was characterized with atomic force microscopy (AFM, Figure S1a). The height profile (Figure S1b) shows a very small variation within 1.5 nm, which has little effect on plasmons and SERS. The dark field image of each Au NP changes to yellow due to the induced coup­ling between the Au NPs and Au film as shown in Figure 1c. A ring-shaped void space between the Au NPs and Au film can be discerned using SEM (Figure 1e-g) although it is difficult to clearly visualize the shape of the entire gap in such semi-embedded nanostructures.

**Figure 1:**
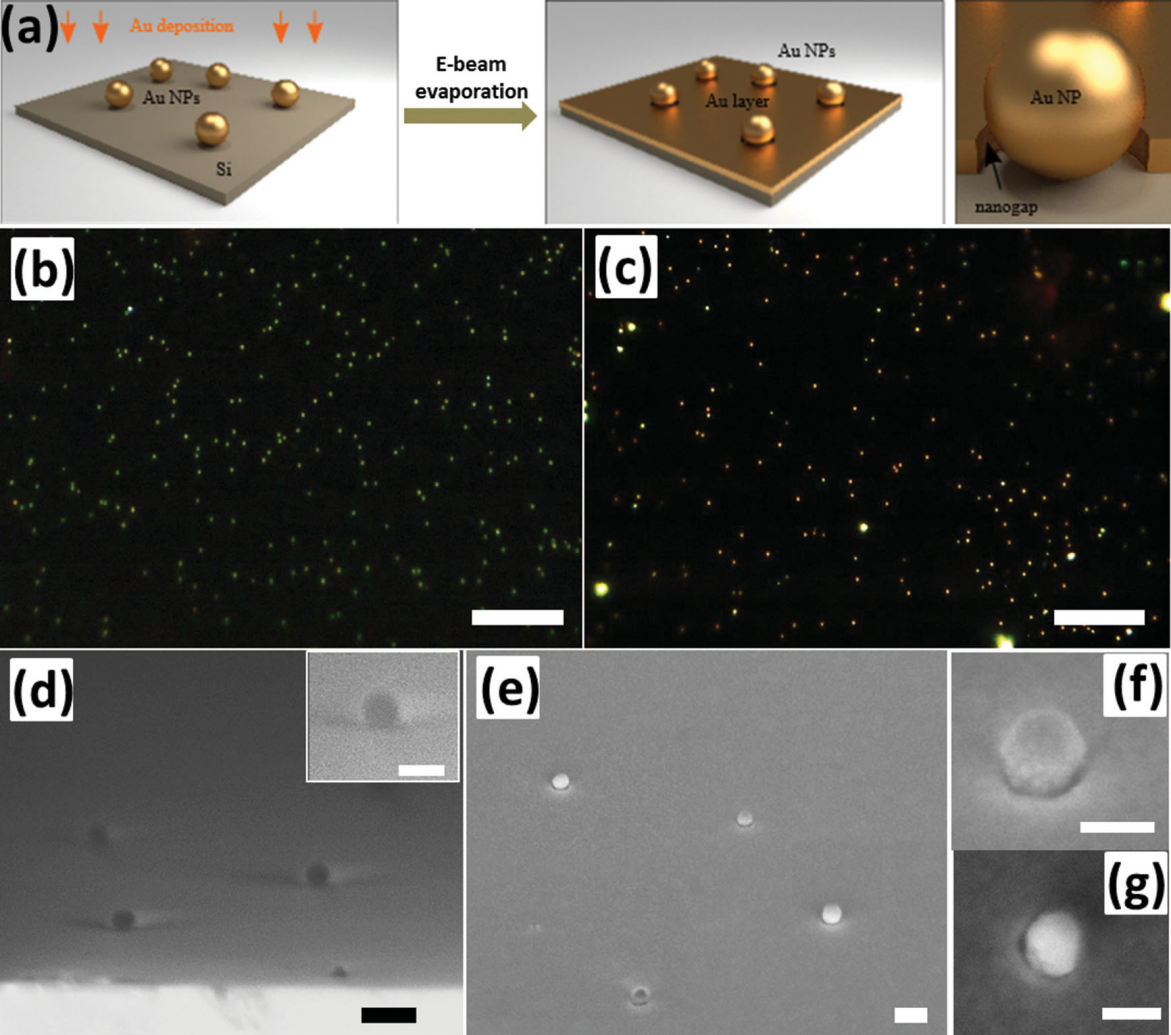
(a) Scheme for generating the nanogaps via colloidal lithography. (b) and (c) are dark field optical images of Au NPs on Si wafer before and after Au evaporation (scale bars, 20 μm). (d) and (e) are the corresponding SEM images (scale bars 200 nm), inset in (d), and (f, g) are the magnified view. (f) 45° tilted, (g) 15° tilted (scale bars 100 nm).

The thickness of the Au films can be adjusted in order to tune the size of the void space. We deposited Au films on the 80 nm Au NPs with thicknesses of 10, 20, 30, 35, 40 and 50 nm. This leads to a change in the color of the Au NPs in dark field optical images from green, to yellow, to red and eventually white as shown in **Figure** **2**a-f. The insets show the corresponding SEM images of the Au NPs, which depict how the gap between the Au NP and Au film gradually decreases with increasing thickness of the Au films. Eventually they merge into a continuous film after the film thickness exceeds 40 nm. The scattering spectra of these Au NPs surrounded with different thickness of Au films are shown in Figure 2g. Each spectrum in Figure 2g is the average of over 20 different Au NPs. The statistics of these spectra is shown in SI Figure S2, with the variation of the peak position being within 25 nm. The observed dominant long wavelength mode corresponds to the longitudinal coupled plasmon mode. The relation of peak position and intensity of the longitudinal mode for different thicknesses of Au films is summarized in Figure 2h. With increasing Au film thickness, the coupling between Au NPs and Au film becomes stronger, as seen in the red-shift of the long­itudinal mode (red solid line). The transverse mode on the other hand remains almost constant at 530 nm (Figure 2g). Once the thickness of Au exceeds 30 nm, however, the rate of red-shift saturates at around 650 nm (Figure 2h, red solid line). This agrees with geometric predictions that the gap should close when the thickness of Au film reaches 40 nm for D = 80 nm Au NPs. It is possible some gaps close but leave nanocavities embedded between the NPs and film which remain unaffected by further Au deposition on top. When the thickness of the Au film reaches 35 nm, for which the smallest gap sizes are expected, the scattering intensity reaches a maximum (black solid line in Figure 2h). Further increase of the thickness of Au film results in the closure of the gap with reduction in plasmonic coupling and hence weaker scattering intensity. We also note that when the film thickness reaches 35 nm, there is a slight blue shift (∼2 nm) of the longitudinal peak compared to 30 nm films, which is possibly due to a slight conductivity across the gap either by tunneling[42] or the formation of few metallic interconnects between the NP and the film due to the finite grain size of the evaporated gold.[43]

**Figure 2:**
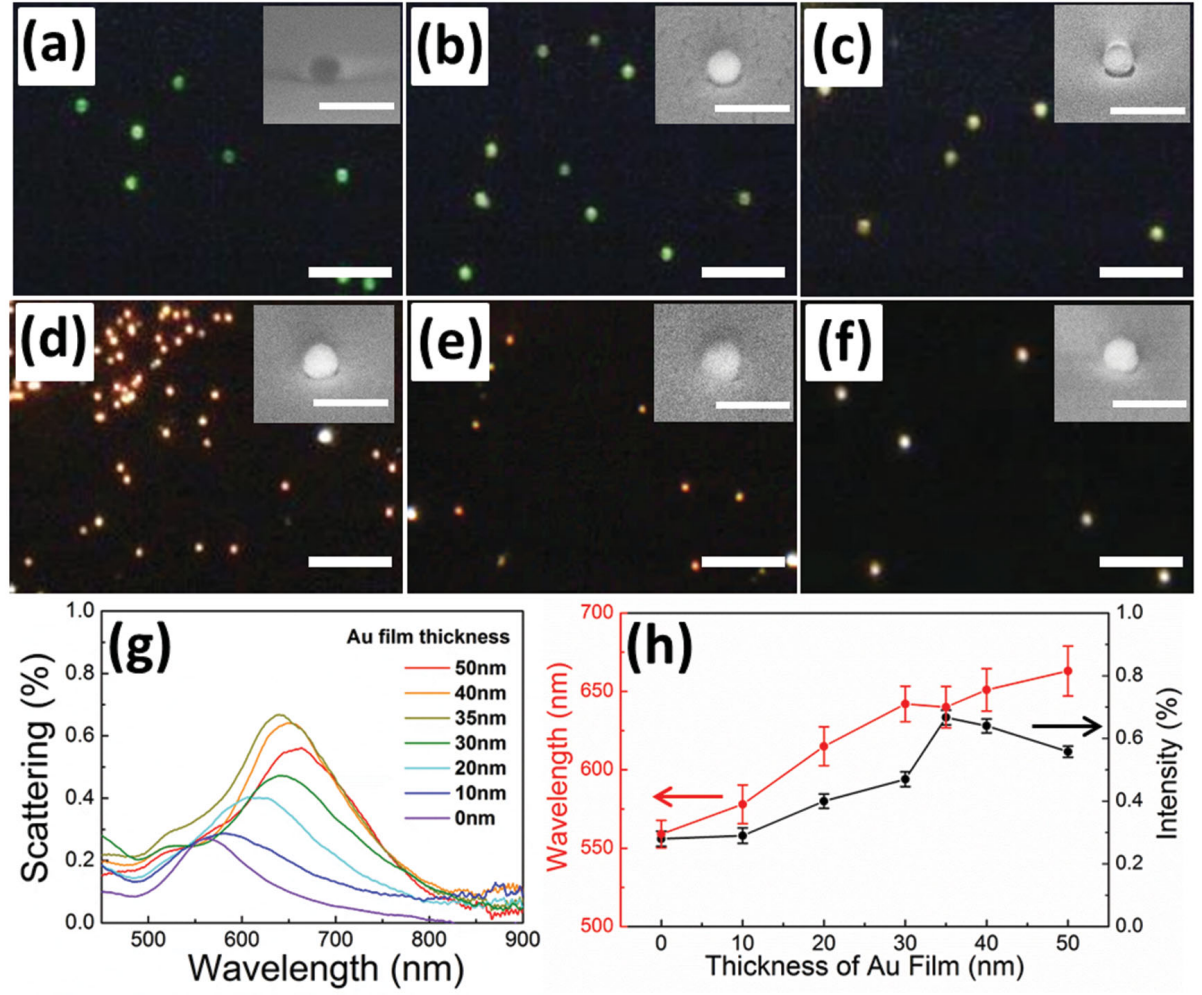
Dark field images of Au NPs on (a) Si wafer, and inside Au films with different thicknesses: (b) 10 nm, (c) 20 nm, (d) 35 nm, (e) 40 nm, and (f) 50 nm (scale bars 5 μm). Insets show the corresponding SEM images (scale bars 200 nm). (g) Scattering spectra of Au NPs with evaporated Au films of different thicknesses. (h) Change of peak position and scattering intensity as a function of Au film thickness.

This geometry of isolated Au NPs in the holes of an infinite Au film was simulated using the finite-difference time-domain (FDTD) method (Lumerical FDTD solutions v8.6). As shown in **Figure** **3**a, with increasing film thickness, the resonant electric field enhancement in the gap between the Au NPs and Au film becomes stronger and reaches a maximum magnitude of approximately 100 for a 40 nm Au film. After that, further deposition of Au leads to the closure of the gap and the electric field enhancement decreases due to conductive contact. The simulated scattering spectra (Figure 3b) and the shift of the resonance (Figure 3c) match relatively well to the experimental spectra in Figure 2g and h. Deviations beyond 40 nm are likely to arise from the real morphology of the Au NP/Au film construct, which is smoothed out compared to the simple combination of thicker Au film and elongated Au NPs used in the simulations.

**Figure 3:**
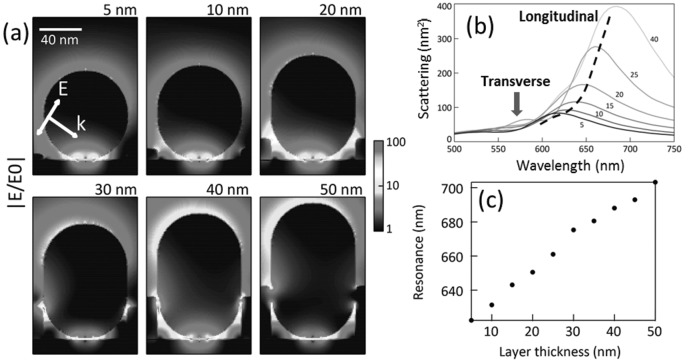
Finite-difference time-domain simulations of the nanogaps between the Au NPs and Au film. (a) Resonant electric field profile of Au NP coupled with different thicknesses of Au film from 5 to 50 nm under *p*-polarized plane wave illumination incident at 55°. (b) Scattering intensity under unpolarized plane wave illumination at 55° and collected over a cone with numerical aperture NA = 0.8, to replicate the experimental dark-field configuration. (c) Shift of the resonance with increasing Au film thickness.

The strong coupling between the Au NPs and Au film in these ring-shaped nanogaps provides a clean and accessible substrate for SERS detection. To verify its performance, the substrate is immersed in thiophenol ethanol solution (10 mM) for 5 mins, rinsed with clean ethanol, and blow dried under nitrogen. The SERS measurements are acquired with a Renishaw inVia Raman microscope based on averaging 5 randomly picked nanoparticles (**Figure** **4**a). The Raman intensity of the 1072.6 cm^−1^ peak is extracted for quantitative comparison (see Figure 4b). Clearly, there is a dramatic increase of the intensity when the film thickness reaches 30 nm. With a thickness of 35 nm, the Raman signal shows the strongest enhancement (150 counts/mW/s) because of the very small nanogap (<5 nm) that is present in this situation. We assume thiophenol is a cylindrically shaped molecule with a length (*l*) of 0.9 nm and a diameter (2*r*) of 0.6 nm. Therefore the contact area of each thiophenol molecule is *A_mol_* = *πr*^2^ = 0.28 nm^2^. We ignore the negligible enhancement arising from the flat Au films and from the top of Au NP when *h* = 0, 10, 20, 30, and 35 nm, so in these cases the effective SERS areas (A*_SERS_*) for the thiophenol molecules are *A*_SERS_ = 2*πR*^2^+2*πRh* = 251(40+*h*) nm^2^, while for 40 and 50 nm thick films, because the gap is closed and lightning rod effect takes a role, the active areas are *A*_SERS_ = 2*πR*^2^ = 1 × 10^4^ nm^2^. We assume the Au is fully covered with a monolayer of thiophenol, so the number of molecules contributed to SERS is N*_mol_* = *A_SERS_*/*A_mol_*. Hence the single molecule Raman intensity at 1072.6 cm^−1^ is S/N*_mol_* counts/mW/s/molecule. The Raman intensity of neat thiophenol (∼9.8 M) is 1372 counts/mW/s at 1092.7 cm^−1^ (Figure S5) which is the original C-S stretching peak seen in SERS at 1072.6 cm^−1^ (this downshift is possibly due to a significant portion of S movement),[44] with effective laser spot volume of around 523 μm^3^, which would include 5.1 × 10^8^ thiophenol molecules. Hence the Raman intensity from each molecule is 2.7 × 10^−6^. By using this value for normalisation, the enhancement factor for different film thickness are then 53, 178, 172, 784, 851, 145 and 225, respectively for *h* = 0, 10, 20, 30, 35, 40 and 50 nm. As expected, the smallest gaps around *h* = 35 nm show the largest enhancements. The actual enhancement factor should be even higher since the thiophenol molecules absorbed on the Au surface never reaches a fully covered monolayer with only 5 mins immersion.

**Figure 4:**
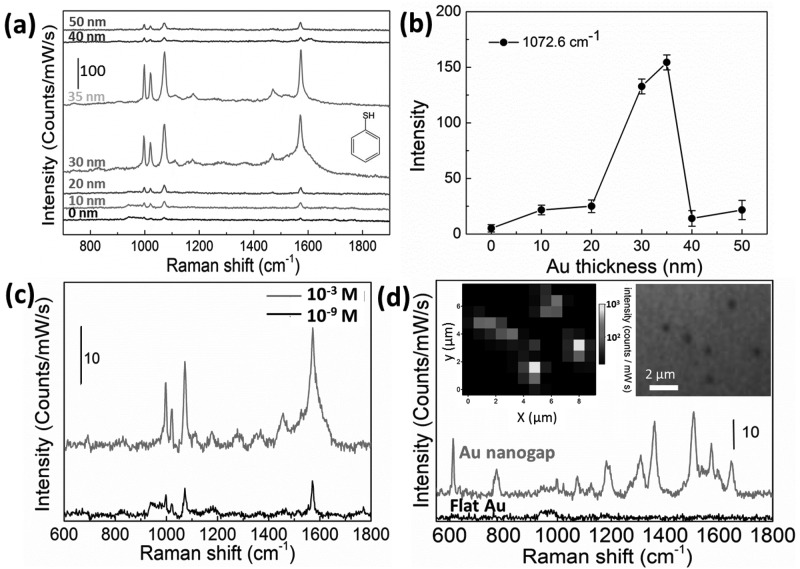
(a) SERS spectra of thiophenol measured with Au film of different thickness. (b) Relation between the SERS signal intensity of peak at 1072.6 cm^−1^ and different thickness of Au film deposited. (c) SERS spectra of thiophenol at different concentrations detected from nanogaps formed with Au film thickness of 35 nm. (d) SERS spectra of R6G (10^−6^ M) measured at the Au nanogaps and control flat Au films. Insets are the SERS mapping and corresponding optical image.

The main limitation to further enhancement is the inhomogeneity in the deposition around the nanoparticles, as well as the diameter of the NPs, which leads to a range of gap sizes. Further increases of the thickness then lead to the closure of the gap reducing the enhancement factor to typical single particle levels. Although it is difficult to measure the precise size of such small gaps, the well-controlled deposition procedure simultaneously tracked through quartz crystal balance (<5% thickness error) enables theoretical estimation of the gap sizes based on the film thickness (Figure S3). The trend of SERS amplitudes thus correlates well with the size of the gap. Such a tailorable SERS substrate is applicable for the detection of a range of Raman active molecules over a broad range of concentrations from 10^−3^ to 10^−9^ M (Figure 4c). To demonstrate its applicability as a general SERS platform beyond the use of molecules with strong Au binding affinities (such as thiolated molecules), we additionally performed SERS measurement on Rhodamine 6G (R6G) (Figure 4d). Here too, the nanogap region clearly gives a strong SERS signal while almost no peaks can be identified on flat Au films. To access the uniformity of the SERS substrate, a Raman map was acquired (insets of Figure 4d). The Raman signal intensity profile (1570 cm^−1^) in the map (left) matches well with the location of the nanoparticles in the optical image (right), which further confirms that such enhancement originates only from the hot spot in the nanogaps. The variation of SERS intensity is likely a consequence of the shape variation of the Au NPs. The morphology of the nanogap is also potentially addressable electrically through the underlying Si substrate, opening up potential for individual bias tuning of such plasmonic nanogaps.

In summary, SERS active nanogaps were fabricated through colloidal lithography based on Au NPs. The Au NPs act as a mask to shadow a ring-shaped region for the e-beam evaporation, thereby enabling fabrication of nanogaps between the Au film and the Au NPs. The gap size is tuneable via the thickness of the evaporated Au film. When the gap size decreases, the longitudinal resonance between the Au NPs and Au film shows a strong red shift and increase in scattering intensity. The Raman signals of thiophenol are dramatically enhanced for smaller gaps, and show optimal enhancement at 35 nm just before closure of the gap. Our nanogap motif is thus a simple but highly effective plasmonic nanostructure and can serve as low-cost and large area SERS substrate for diagnostics and sensing. Furthermore, it enables mapping of individual nanoparticle constructs, thereby opening possibilities for few-to-single molecule spectroscopies.

## Experimental Section

*Substrate Fabrication*: 20 μL of 80 nm Au NP aqueous dispersion (obtained from BBI) was drop cast on oxygen plasma cleaned Si wafers. After 5 minutes, the dispersion was rinsed away with DI water, leaving a sparse coverage of isolated Au NPs on the Si wafer. The Au film evaporation was carried out with a Lesker e-beam evaporator at voltage of 10 kV under a vacuum of 4 × 10^−6^ mBar. The deposition rate was 1 Å/s.

*Microscopy*: Optical dark-field images were captured in an Olympus BX51 upright microscope using a 100× dark field objective (Olympus LMPLFLN-BD, NA 0.8). Scattered light from single NPs was collected through a confocal 50 μm fiber and sent to an Ocean Optics spectrometer (QE65000) for spectral analysis. The SEM images were obtained on a LEO 1530VP (Zeiss) at an accelerating voltage of 5 kV. The SERS measurements were performed on a Renishaw inVia Raman microscope with a 633 nm excitation laser (power 1 mW) and an integration time of 30 s. For SERS of trace concentrations of thiophenol, 10 mW laser power was applied with integration time of 60 s. The SERS mapping of R6G was done with a 100× objective (NA = 0.85) with a sampling step of 0.8 μm. For each pixel of the map a full Raman spectrum was recorded. From the individual spectra the baseline corrected intensity at 1570 cm^−1^ was extracted and plotted as function of the position on the sample.
