# Supplementary material for: Self-Aligned Colloidal Lithography for Controllable and Tuneable Plasmonic Nanogaps
Source: Small. 2014 Dec 15;11(18):2139–43. doi: 10.1002/smll.201402639 (PMC4515099; doi:10.1002/smll.201402639)
Supplement: Supplementary file 1 [file smll0011-2139-sd1.pdf]

Copyright WILEY-VCH Verlag GmbH & Co. KGaA, 69469 Weinheim, Germany, 2014.

Supporting Information

for *Small*, DOI: 10.1002/smll.201402639

Self-Aligned Colloidal Lithography for Controllable and Tuneable Plasmonic Nanogaps

Tao Ding, Lars O. Herrmann, Bart de Nijs, Felix Benz, and Jeremy J. Baumberg**

Copyright WILEY-VCH Verlag GmbH & Co. KGaA, 69469 Weinheim, Germany, 2013.

Supporting Information

Self-aligned colloidal lithography for controllable and tuneable plasmonic nanogaps

Tao Ding, Lars O. Herrmann, Bart de Nijs, Felix Benz, and Jeremy J. Baumberg**

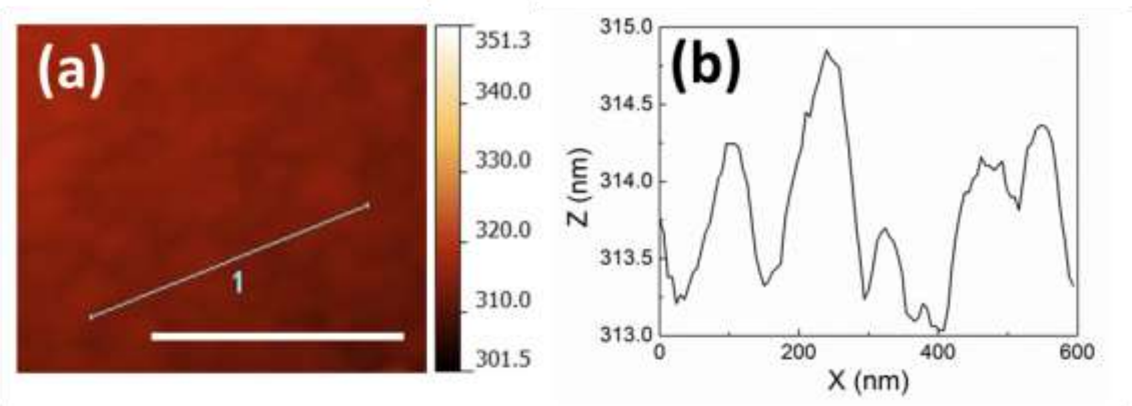

Figure S1. (a) The AFM image of evaporated Au films and (b) their surface height profile. Scale bar is 5 μm.

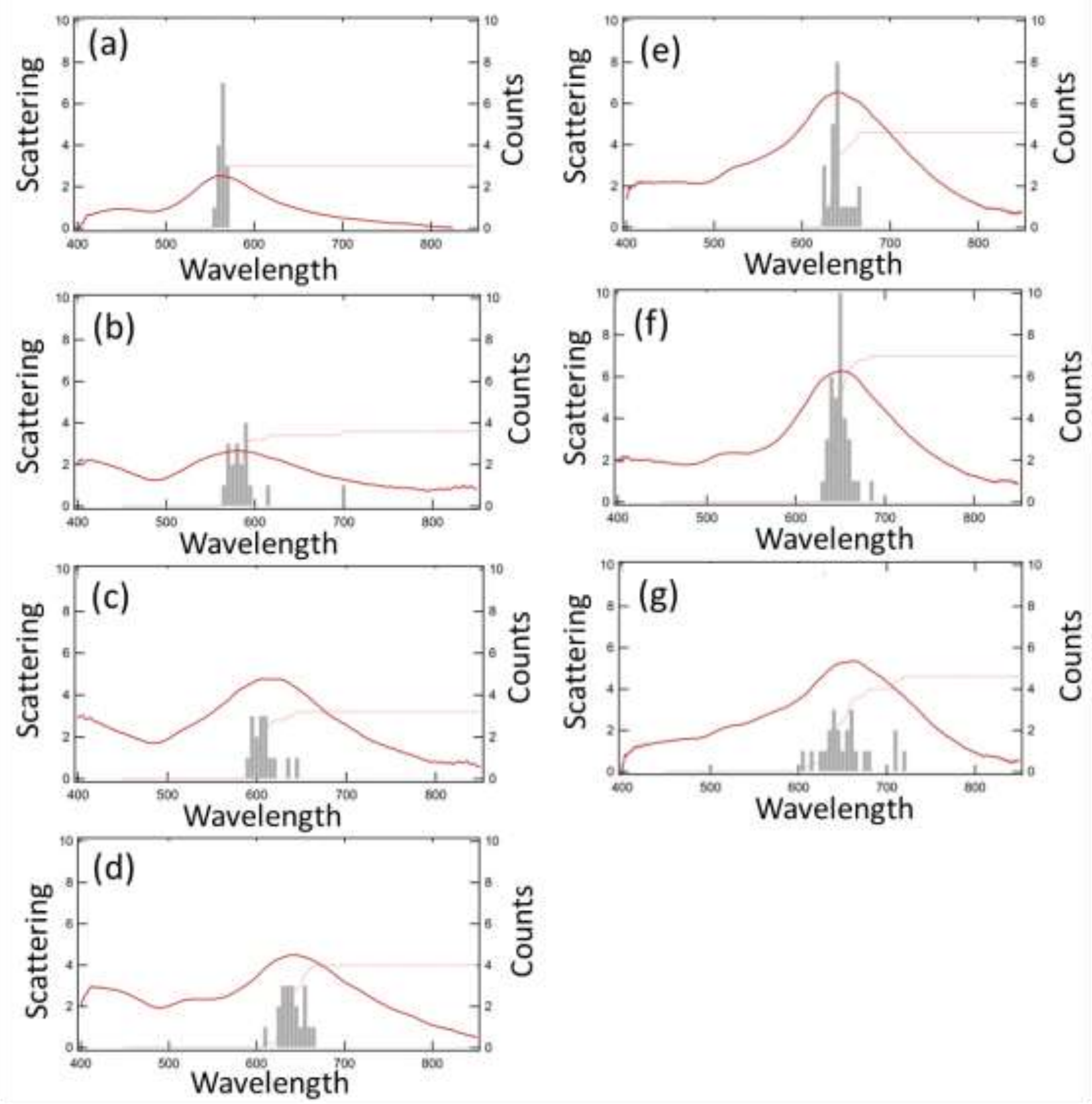

Figure S2. Statistics of scattering spectra of Au NPs surrounded by Au films with a ring-shaped gap in-between. The thickness of the Au film is (a) 0 nm (b) 10 nm (c) 20 nm (d) 30 nm, (e) 35 nm, (f) 40 nm, (g) 50 nm and the substrate underneath is a Si wafer. The red solid line is the average of all the spectra taken from approximately 20 different Au NPs. The grey bar charts are the corresponding histograms of the longitudinal peak positions. The light red line shows the accumulation of the counts.

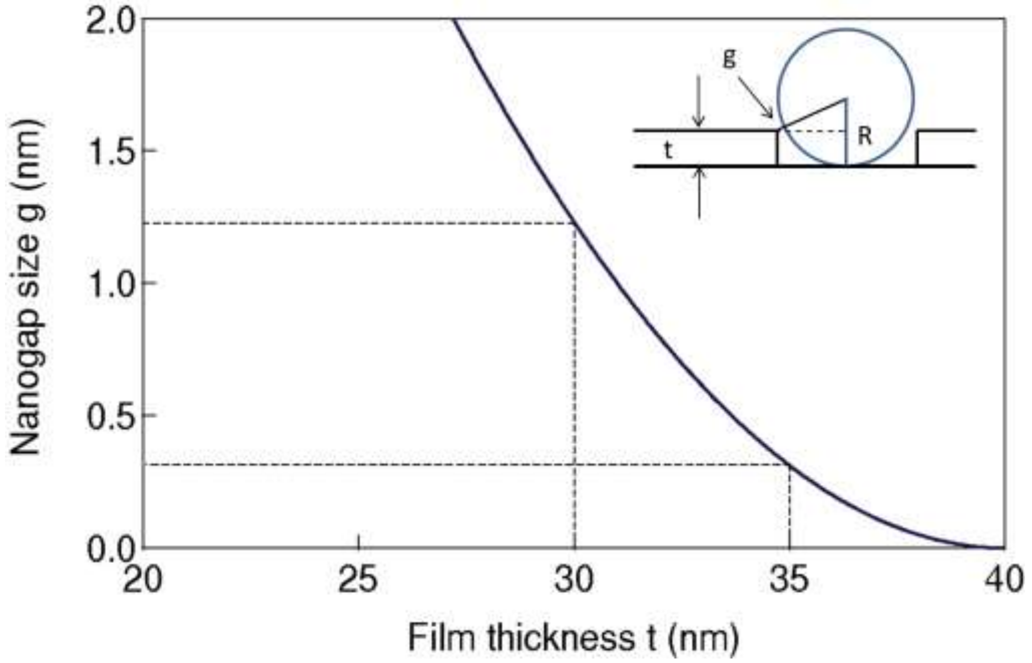

Figure S3. Theoretical size of nanogap as a function of gap thickness for a Au NP with diameter of 80 nm.

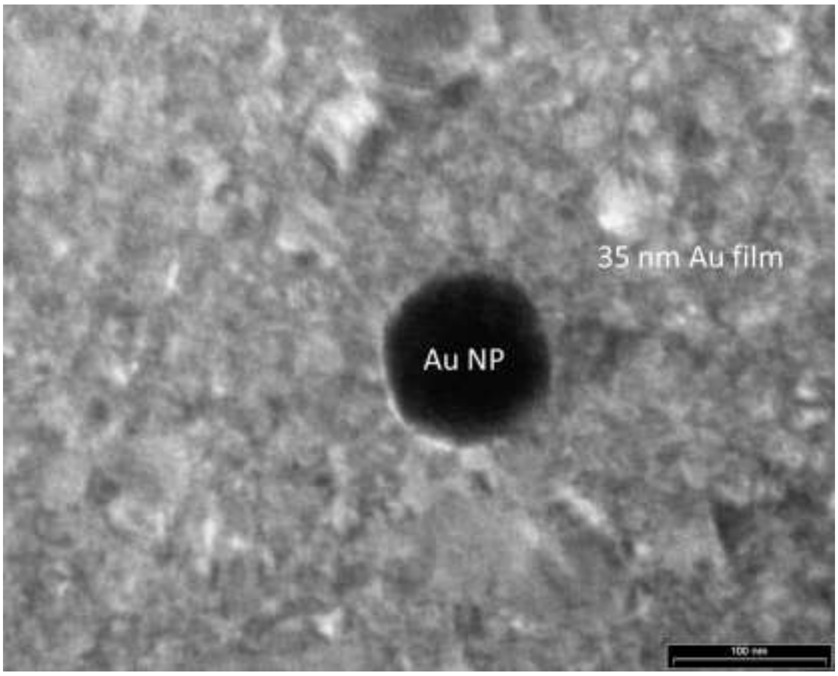

Figure S4, TEM image of Au NP (80 nm) on the cabon substrate evaporated with 35 nm Au films.

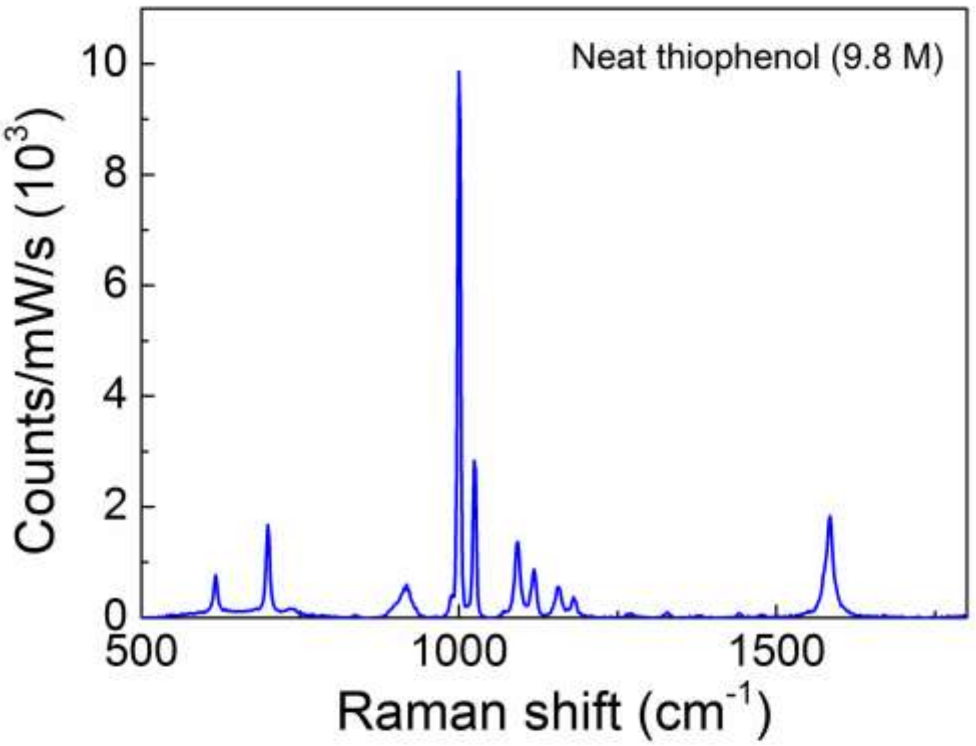

Figure S5. Raman spectrum of neat thiophenol (9.8 M).
